# Nitric oxide-primed engineered extracellular vesicles restore bioenergetics in acute kidney injury via mitochondrial transfer

**DOI:** 10.7150/thno.113741

**Published:** 2025-04-13

**Authors:** Fei Peng, Xiaoniao Chen, Lingling Wu, Jiayi He, Zongjin Li, Quan Hong, Qiang Zhao, Meng Qian, Xu Wang, Wanjun Shen, Tingting Qi, Yiyu Huang, Guangyan Cai, Chuyue Zhang, Xiangmei Chen

**Affiliations:** 1 School of Medicine, Nankai University, Tianjin 300071, China.; 2 Department of Nephrology, First Medical Center of Chinese PLA General Hospital, National Key Laboratory of Kidney Diseases, National Clinical Research Center for Kidney Diseases, Beijing Key Laboratory of Kidney Diseases Research, Beijing 100853, China.; 3 Department of Ophthalmology, Third Medical Center of Chinese PLA General Hospital, Beijing 100853, China.; 4 Clinical Research Center, First Affiliated Hospital of Shantou University Medical College, Shantou, Guangdong 515041, China.; 5 Key Laboratory of Bioactive Materials, Ministry of Education, College of Life Sciences, Nankai University, Tianjin 300071, China.; 6 Department of Nephrology and Institute of Kidney Diseases, West China Hospital, Sichuan University, Chengdu 610041, China.

**Keywords:** Mesenchymal stem cells, Extracellular vesicles, Nitric oxide, Mitochondrial homeostasis, Acute kidney injury.

## Abstract

**Background:** The disruption of mitochondrial homeostasis in acute kidney injury (AKI) is an important factor that drives persistent renal dysfunction. Mesenchymal stem cell-derived extracellular vesicles (MSC-EVs) have shown great therapeutic potential in AKI, but insufficient specificity of targeting the impaired mitochondrial function. Herein, we developed an engineered nitric oxide (NO)-primed MSC-EVs (pEVs) to restore mitochondrial homeostasis for AKI therapy.

**Methods:** A cisplatin-induced AKI model was established to investigate the therapeutic effects of MSC-EVs. Proteomic and Western blot analyses compared mitochondrial cargos and functional assays included mitochondrial complex I activity and Adenosine triphosphate (ATP) quantification. Mitochondrial transfer was tracked using flow cytometry and confocal imaging. Mitochondrial dynamics, oxidative stress, and apoptosis were evaluated through ATP measurement, western blotting and rotenone-mediated respiratory chain inhibition.

**Results:** Our data indicated that pEVs outperformed cEVs in restoring renal function and histopathology. Additionally, a reduction in mitochondria-associated oxidative stress and cell death was observed. Proteomic profiling revealed that NO priming enriched pEVs with mitochondrial complex I components, which directly enhanced their bioenergetic capacity, as evidenced by higher mitochondrial complex I activity and elevated ATP production compared to cEVs. In vivo tracking confirmed targeted delivery of pEV-derived mitochondrial contents to renal tubular cells, reducing mitochondrial reactive oxygen species (ROS) and restoring mitochondrial mass. Crucially, mitochondria-depleted pEVs abolished these therapeutic effects, establishing mitochondrial cargos as the primary therapeutic driver. Furthermore, pEVs activated a pro-survival cascade in recipient cells, showing superior efficacy in promoting mitochondrial biogenesis, dynamics, and mitophagy, thereby restoring renal mitochondrial homeostasis.

**Conclusion:** Our study elucidated a mitochondria-targeted therapeutic strategy enabled by engineered EVs that deliver functional cargo to restore mitochondrial homeostasis. These advances provide transformative potential for AKI and other mitochondrial disorders.

## Introduction

Acute kidney injury (AKI) represents a prevalent and severe renal disorder characterized by a rapid decline in kidney function, with global incidence figures indicating approximately 13.5 million cases annually 1. Mitochondrial dysfunction is recognized as a central pathophysiological mechanism in AKI, profoundly affecting cellular bioenergetics and oxidative stress responses 2, 3. Given the pivotal role of mitochondria in maintaining renal homeostasis and energy metabolism, strategies aimed at restoring mitochondrial function have emerged as promising therapeutic avenues for AKI 4, 5.

Despite advancements, the translation of mitochondria-targeted therapies from preclinical models to clinical application poses significant challenges 6. These challenges encompass not only the complexities of mitochondrial biology and bioenergetics but also potential adverse effects associated with targeted interventions 7. Recent studies have highlighted a novel approach involving mitochondrial transfer, which holds potential for enhancing tissue repair and functional recovery in renal injury settings 8. Mechanisms for mitochondria transfer, facilitated by cellular processes such as tunneling nanotubes, gap junction channels, and extracellular vesicles (EVs), have garnered increasing attention for their ability to influence cellular metabolism and improve mitochondrial function beyond the immediate injury site 9.

Mesenchymal stem cells (MSCs) are a type of cell found in various tissues that possess the potential for differentiation and self-renewal, making them promising candidates for promoting kidney tissue repair 10. Recent studies have revealed that MSCs are well-suited carriers for mitochondria because of their lower energy demands and ability to support a healthy mitochondrial output 11, 12. Lin et al. recently reported in *Nature* recently that MSCs facilitate endothelial cell engraftment via mitochondria transfer, offering potential for a new approach for vascular cell therapy 13. However, persistent challenges remain in achieving effective long-distance transport and improving the efficiency of mitochondrial transfer. Therefore, it is necessary to devise innovative strategies to overcome these limitations.

In this study, we propose a novel strategy of nitric oxide (NO)-releasing biomaterials priming of MSCs to generate educated-EVs enriched with mitochondrial contents. We show that these engineered EVs effectively transfer mitochondrial contents to renal cells, thereby improving mitochondrial homeostasis and potentially ameliorating AKI. This approach leverages two key advantages: first, EVs are lipid bilayer nanoparticles that inherit cargo from parent cells, facilitating long-distance mitochondrial delivery without the risks associated with direct MSC transplantation 14, 15. Second, NO functions as a molecular switch, effectively triggering mitochondrial biogenesis across various cell types 16, 17. Despite its well-established role, the application of NO in enhancing EV therapy to improve therapeutic outcomes in AKI remains underexplored. This innovative approach underscores the potential of NO-primed strategies in improving EV-based therapies for diseases associated with mitochondrial dysfunction.

## Material and methods

### Measurement of NO release *in vitro*

The cumulative release of NO from Chitosan (CS)-based nitric oxide (NO)-releasing biomaterials (CS-NO) was further assessed using NO-specific DAF-2 fluorescent probe (MX4705, Maokang Biotechnology, China) assays. After incubating at 37°C for 24 h, the release of NO in phosphate-buffered saline (PBS) was measured as the fluorescence intensity (excitation at 485 nm, emission at 538 nm) of the NO-specific probe DAF-2 (10 μM). The results are expressed as the fold change compared with PBS.

### Cell culture

#### MSC and CS-NO preconditioning

Human placenta-derived MSCs were purchased from the National Engineering Research Center of Cell Products. Human placenta-derived MSCs from a single donor were isolated using collagenase II (Gibco, Grand Island, NY) and trypsin (Gibco) digestion following a previously described protocol 18. The isolated cells were cultured in Dulbecco's modified Eagle medium/nutrient mixture F-12 (DMEM/F12, Gibco), supplemented with fetal bovine serum (FBS 10%, Gibco), penicillin (100 U/mL; Gibco), and streptomycin (100 μg/mL; Gibco). For NO stimulation, complete DMEM/F12 medium was supplemented with CS-NO and β-galactosidase to stimulate NO production. CS-NO is a glycosylated NO compound formed by conjugating N-diazeniumdiolates as NO donors with chitosan (CS) to create a comb-shaped polymer that releases NO upon the addition of glycosidase at specific concentrations 18-21.

#### Renal epithelial cells (HK-2)

HK-2 cells (human proximal tubular cell line) were cultured in DMEM/F12 (1:1) with 10% FBS and 100 U/mL penicillin-streptomycin (Gibco) and passaged every 2 days. The cells were grown at 37 °C in a humidified 5% CO_2_ atmosphere. To mimic cisplatin- induced kidney injury in vitro, cisplatin was used to treat HK-2 cells for 24 h. Then, HK-2 cells were incubated with 100 μg/mL EVs. The viability of HK-2 cells was detected by Cell Counting Kit-8 (CCK-8) (CK-04, Dojindo, Japan).

### EV isolation

To obtain EVs derived from MSCs, FBS (EXO-FBS-50A-1, SBI System Biosciences, USA) devoid of EVs was used to culture MSCs in a medium with or without CS-NO. The EVs were subsequently isolated from the culture supernatant via ultracentrifugation. The freshly obtained culture supernatant was initially centrifuged at 300 ×g for 5 min at 4°C and at 2,000 ×g for 10 min, followed by centrifugation at 10,000 ×g for 30 min to eliminate cell debris. Next, the supernatant was filtered through 0.22 μm filters into new tubes and centrifuged at 130,000 ×g for 80 min at 4°C. Subsequently, the supernatant was discarded, and the pellet was washed with 30 mL of PBS. This was followed by a second ultracentrifugation at 130,000 ×g for 2 h at 4°C. The resulting supernatant was discarded, and the EVs were resuspended in 100 μL of PBS 22, 23.

### Transmission electron microscopy (TEM)

Fresh MSC-EVs and kidney tissues were fixed with 2.5% glutaraldehyde. Subsequently, 10 μL of the fixed sample was incubated on a carbon-coated formvar film on a copper grid for 10 min. After washing with distilled water, the samples were treated with 1% uranyl acetate for 5 min and dried in a clean dish for TEM analysis. Imaging was performed using a FEI TF20 transmission electron microscope at 200 kV.

### MSC-EV size determination by dynamic light scattering (DLS) and Nanoparticle tracking analysis (NTA)

The size of MSC-EVs was quantified by DLS on a Nanosizer instrument. Prior to analysis, the EV samples were diluted in 1 mL of PBS and loaded into the instrument, which enabled automated monitoring and determination of particle size.

NTA of cEVs or pEVs samples was performed using a ZetaView® PMX 120 instrument (Particle Metrix, Germany) with laser scattering configured at 520 nm. EVs were diluted 1:2000 in particle-free ultrapure water to achieve optimal scattering intensities. Particle size distribution and concentration profiles were acquired under standardized detection parameters (minimum area 5, maximum area 1000, and minimum brightness 20) at 25°C.

### EV quantification

cEVs or pEVs protein concentrations were determined via the bicinchoninic acid (BCA) assay using Pierce^™^ BCA Protein Assay Kits (Thermo Fisher Scientific, #23225) according to the manufacturer's protocol. Briefly, EV lysates were prepared and quantified against a standard curve with absorbance measured at 562 nm.

### Animals and treatment

Male C57BL/6J mice (18-22 g, 8 weeks old) were obtained from SPF (Beijing, China) Biotechnology Co. The mice were housed in a controlled environment with consistent temperature and humidity, and alternating 12 h light/dark cycles. This research plan was approved by the Animal Ethics Committee of the PLA General Hospital before the start of the experiment (Approval No.: 2019-X15-65). All animal care and experimental procedures complied with the guidelines for the Care and Use of Laboratory Animals published by the United States National Institutes of Health (NIH publication, 2011 Revision).

AKI was induced in the experimental group via a single intraperitoneal injection of 20 mg/kg cisplatin (Hansoh Pharma, Sigma, USA), while the control group received an equivalent volume of normal saline via intraperitoneal injection. Control (c) EVs, EVs derived from CS-NO-primed MSCs (pEVs), or PBS (100 μg/dose for cEVs or pEVs; n = 5 for each group) were administered through the tail vein for intervention. On day 3, the mice were euthanized under anesthesia, and their blood was collected and centrifuged at 3,000 rpm for 10 min to obtain serum samples. The kidney tissues were immediately frozen in liquid nitrogen or fixed with 4% formaldehyde after euthanasia.

### Biodistribution and intracellular uptake of EVs

cEVs or pEVs (200 μg) dissolved in 1 mL of PBS were incubated with 4 μM Aggregation-Induced Emission (AIE) dye (Tingo Red, Tingocell, Tianjin, China). The mixture was then ultracentrifuged at 130,000 ×g for 2 h to remove unbound dye. Subsequently, the aggregates of AIE-EV aggregates were resuspended in PBS. The protein concentrations of all analyzed EVs were quantified using a bicinchoninic acid (BCA) assay (Sangon Biotech, Shanghai, China) following the manufacturer's protocol. AIE-EVs were used for all subsequent experiments. AKI mice were randomly divided into two groups and injected intravenously (100 μg per mouse). At selected time points after administration, the mice were anesthetized and imaged non-invasively using an IVIS Lumina Imaging System (Xenogen Corporation, Hopkinton, MA, USA). To investigate the biodistribution of AIE-EVs in AKI mice, AIE signals from cEVs and pEVs from harvested and sliced kidneys and other organs were examined using the IVIS Lumina Imaging System. Concurrently, to further study the in vivo fate and cellular distribution of the injected EVs, the collected kidneys were fixed and sectioned into 5 μm cryosections for immunostaining. For AIE, λem = 630 nm and λex = 488 nm. The uptake was observed using fluorescence microscopy.

### Histological analysis

The fixed kidney tissues were embedded in paraffin and then cut into 3-5 μm sections. These sections were stained using the periodic acid-Schiff (PAS) or immunohistochemistry technique. In the PAS-stained samples, eight non-overlapping visual fields (200 × magnification) of the outer medulla of the kidneys were randomly selected to evaluate renal damage. Tissue damage, including loss of brush border, cast formation, cell necrosis, and tubule dilation, was quantified using the acute tubular necrosis (ATN) score to assess tubular injury, as described in previous reports 23. Apoptotic cells were detected by a terminal deoxynucleotidyl transferase-mediated dUTP nick-end labeling (TUNEL) assay using commercial kits (Beyotime, Beijing, China) according to the manufacturer's instructions.

### Renal function analysis

To assess the residual function of the injured kidney, serum samples were collected to evaluate renal function markers, including blood urea nitrogen (BUN) and serum creatinine (SCr). Concentrations of BUN and SCr were measured using BUN and SCr assay kits (Nanjing Jiancheng Bioengineering Institute), respectively.

### Western blotting (WB)

A BCA protein quantitation assay (Thermo Fisher Scientific, Waltham, MA, USA) was used to measure the protein levels in tissue or cell lysates and EVs. After denaturation, the proteins were transferred onto nitrocellulose membranes (Roche, Switzerland). The membranes were then blocked with 5% bovine serum albumin in Tris-buffered saline with 0.1% Tween® 20 detergent at room temperature, followed by incubation with antibodies against Alix (1:1000, WL03063, Wanleibio), CD9 (1:1000, ab92726, Abcam), TSG101 (1:1000, ab125011, Abcam), GM130 (1:1000, ab52649, Abcam), KIM1 (1:1000; ab302932, Abcam), NGAL (1:1000; PA5-79590, ThermoFisher), BAX (1:1000; ab110333, Abcam), BAX (1:1000; ab7977, Abcam), BCL-2 (1:1000; ab182858, Abcam), Active-caspase3 (1:1000; AC033, Bryotime biotechnology), NRF1 (1:1000; 46743S, Cell signaling technology), TFAM (1:1000; 23996-1-AP, Proteintech), ATPB (1:1000; 17247-1-AP, Proteintech), NDUFA13 (1:1000; ab110240, Abcam), PDHA1 (1:1000; 10986-1-AP, Proteintech), LETM1 (1:1000; 16024-1-AP, Proteintech), SDHB (1:1000; 10620-1-AP, Proteintech), COX IV (1:1000; 66110-1-Ig, Proteintech), PDH (1:1000; ab110333, Abcam), p-AMPK (1:1000; 2535, Cell signaling technology), DRP1 (1:1000; ab184247, Abcam), MFN2 (1:1000; ab124773, Abcam), OPA1 (1:1000; 80471S, Cell signaling), PINK1 (1:1000; ab23707, Abcam), PARKIN (1:1000; ab77924, Abcam), LC3 (1:1000; ab192890, Abcam), P62 (1:1000; ab109012, Abcam) and GAPDH (1:10000, 60004-1-Ig, Proteintech) overnight at 4 °C. After washing with TBST, the membranes were incubated with horseradish peroxidase-conjugated goat anti-rabbit or anti-mouse IgG for 2 h at room temperature. Subsequently, the membranes were exposed to Enhanced Chemiluminescence Plus and images were captured using the Bio-Rad ChemiDoc Touch analysis system.

### Reverse transcription-quantitative polymerase chain reaction (RT-qPCR)

Total RNA was isolated from kidney tissues using TRIzol reagent (Thermo Fisher Scientific, Waltham, MA, USA) following the manufacturer's instructions. Subsequently, 1 µg of total RNA was reverse-transcribed to cDNA using a cDNA synthesis kit (E6560S; New England Biolabs, Frankfurt, Germany). RT-qPCR was performed using SYBR Green Master Mix (Applied Biosystems, Foster City, CA, USA) with the primer sequences provided in Supplementary Table S1. The results were visualized using a PCR Detection System (Bio-Rad, USA). Data were normalized to the expression level of 18S rRNA, and relative gene expression was determined using the comparative threshold cycle method.

### Immunostaining analysis

For immunofluorescence (IF) staining, 6 μm sections were returned to room temperature and washed in PBS. Next, the sections were incubated with 5% BSA containing 0.1% Triton X-100 at room temperature for 15 min and then incubated with primary antibodies against KIM1 (1:200; AF1817, R&D), Active-Caspase3 (1:200; WL02117, Wanleibio), mtTFA (1:100, ab30702, Abcam), COX IV (1:100; 66110-1-Ig, Proteintech), TSG101 (1:1000, ab125011, Abcam), NDUFA13 (1:1000; ab110240, Abcam), PDHA1 (1:1000; 10986-1-AP, Proteintech), LC3 (1:100; ab192890, Abcam), and P62 (1:100; ab109012, Abcam) overnight at 4°C. The following secondary antibodies were used: Cy3-or fluorescein isothiocyanate (FITC)-labeled donkey anti-rabbit, anti-mouse, or anti-goat antibodies (1:400; Invitrogen). FITC-labeled Lotus tetragonolobus lectin (LTL, 1:400, Vector Laboratories, Burlingame, CA, USA) was used to stain the proximal tubules. DAPI (ab104139; Abcam) was used for nuclear counterstaining.

For immunohistochemical (IHC) staining of kidney tissues, dewaxing of paraffin-embedded sections, incubation in 3% hydrogen peroxide, and antigen retrieval by microwave heating were performed. The sections were then incubated with primary antibodies against 8-OHdG (1:50; sc-66036, Santa Cruz Biotechnology), mtTFA (1:1000, ab30702, Abcam), and MFN2 (1:1000; ab124773, Abcam) overnight at 4°C. IHC was conducted using Histostain-SP Kits (PV-9001, ZSGB-BIO) and DAB peroxidase substrate kits (ZLI-9017, ZSGB-BIO), following the manufacturer's instructions.

### Measurement of antioxidant enzymes

To assess the levels of oxidative stress in the kidney, the following procedures were performed: 100 mg kidney tissue samples were processed and dissected at 4°C, then immediately snap-frozen in liquid nitrogen. The frozen kidney tissues were stored at -80°C. Subsequently, the fresh kidney tissues were homogenized in pre-cooled PBS and centrifuged at 10,000 ×g for 10 min. The supernatant of the kidney tissues was analyzed using malondialdehyde (MDA), superoxide dismutase (SOD), and catalase (CAT) biochemical kits (Jiancheng, Nanjing, China) following the manufacturer's instructions. The absorbance values were determined using a microplate reader.

### Flow cytometry analysis of active mitochondrial content transfer

The Mito-Tracker Deep Red FM (MTDR) (C1032, Beyotime, China) was introduced into MSCs at a concentration of 10 μM for 50 min to label mitochondrial contents. MTDR-labeled EVs encapsulating mitochondrial contents were isolated from the culture supernatant using ultracentrifugation according to a previously reported method. The mitochondria of HK-2 cells were labeled using Mito-Tracker Green (MTG) (C1048, Beyotime, China). Subsequently, the isolated EVs were co-incubated with HK-2 cells after cisplatin stimulation for 8 h to observe fluorescence. Nuclei were labeled with DAPI (ab104139, Abcam, USA). Additionally, the fluorescence intensity ratio of MTDR to MTG was calculated by flow cytometry.

### Proteomic analysis of MSC-EVs

cEVs or pEVs were washed with PBS and lysed via sonication in 30 µL (minimum workable volume) of lysis buffer. The digested peptide mixtures were subjected to nanospray ionization with positive ion polarity and analyzed using an Orbitrap Fusion Lumos mass spectrometer interfaced with an Easy-nLC 1000 nanoflow liquid chromatography system (Thermo Fisher Scientific). The samples were dissolved in Solvent A (0.1% formic acid in water), loaded onto a homemade trap column packed with C18 reverse-phase resin, and separated using a gradient of 7-32% mobile phase B over 60 min. Mass spectrometric analysis was performed in Data Dependent Acquisition mode with full scans acquired using an Orbitrap mass analyzer at a resolution of 120,000. The most intense ions were selected in the top-speed mode, fragmented via high-energy collisional dissociation, and detected in the Orbitrap at a resolution of 15,000. The dynamic exclusion time was set as 30 s.

Differentially expressed proteins (DEPs) among the different samples were defined using the "limma" package in R software (version 3.10.3) with the criteria of |log fold change (FC)| > 1.2 and p < 0.05 24. Functional enrichment analyses were performed using Gene Ontology (GO; http://www.geneontology.org/), the Kyoto Encyclopedia of Genes and Genomes (KEGG; http://www.genome.jp/kegg/) and Reactome pathway database (http://www.reactome.org/). The Molecular Signatures Database (MSigDB) was used for Gene Set Enrichment Analysis (GSEA v4.3.2; Broad Institute; http://www.broadinstitute.org/gsea/msigdb/index.jsp) 25. The mass spectrometry proteomics data have been deposited to the ProteomeXchange Consortium via the iProX partner repository 26, 27 with the dataset identifier PXD048626.

### ATP measurement

Cellular and MSC-EVs ATP levels were quantified using a bioluminescence-based ATP detection assay (Beyotime Biotechnology, Cat. No. S0027). Briefly, treated cells were harvested and lysed in 200 μL of ATP-specific lysis buffer. Lysates were centrifuged at 12,000 ×g for 5 min at 4°C to pellet insoluble debris, and the resulting supernatants were transferred to fresh tubes. For ATP measurement, 20 μL of supernatant or EVs lysates were combined with 100 μL of luciferase reagent to initiate the ATP-dependent luminescent reaction. Bioluminescence signals were immediately recorded using a microplate reader (Tecan Group Ltd., Switzerland), and ATP concentrations were calculated against a standard curve (0.1-10 μM ATP).

### Mitochondrial complex I activity assay

Mitochondrial complex I enzymatic activity was evaluated in freshly isolated MEC-EVs and cells using a colorimetric assay kit (Abbkine, Cat# KTB1850, Wuhan, China). The activity of the complex I was assessed by colorimetry using commercial kits (Abbkine, Cat. No: KTB1850) according to the manufacturer's instructions. Absorbance measurements at 340 nm were performed on a microplate reader following the manufacturer's standardized protocol.

### Isolation of mito_ex_-pEVs from Mitochondrial-Respiration-Inhibited CSNO-MSCs

To inhibit mitochondrial respiration, CS-NO-primed MSCs were treated with 0.1 μM rotenone (Selleck Chemicals, Cat# S2348), a potent and selective inhibitor of mitochondrial complex I, for 24 h under standard culture conditions (37°C, 5% CO₂). Rotenone blocks electron transport at complex I, thereby suppressing ATP production. After treatment, cell culture supernatants were collected and processed to isolate pEVs depleted of mitochondria (mito_ex_-pEVs) through sequential centrifugation and ultracentrifugation.

### Mitochondrial ROS measurement

Mitochondrial reactive oxygen species (ROS) levels were quantified using MitoSOX^™^ Red (M36008, Thermo Fisher Scientific), a fluorescent probe specific for superoxide anion in mitochondria. Following experimental treatments, cells were incubated with 5 μM MitoSOX^™^ Red in serum-free medium at 37°C for 15 min under light-protected conditions. After incubation, cells were washed three times with PBS to remove unbound dye. Stain the cells with counterstains as desired and mount them on a warm buffer slide for fluorescence imaging.

### Statistical analysis

The data were analyzed using GraphPad Prism 7.0, and all group data are presented as the mean ± SEM. The statistical analyses were conducted using Student's *t*-test or one-way analysis of variance (ANOVA). Column means were compared using one-way ANOVA with treatment as the independent variable. When the ANOVA showed a significant difference, post-hoc analysis between group means was performed using Tukey's or the uncorrected Fisher's LSD multiple comparison test. Statistical significance was set at p < 0.05.

## Results

### Generation and characteristics of pEVs

**Figure 1:**
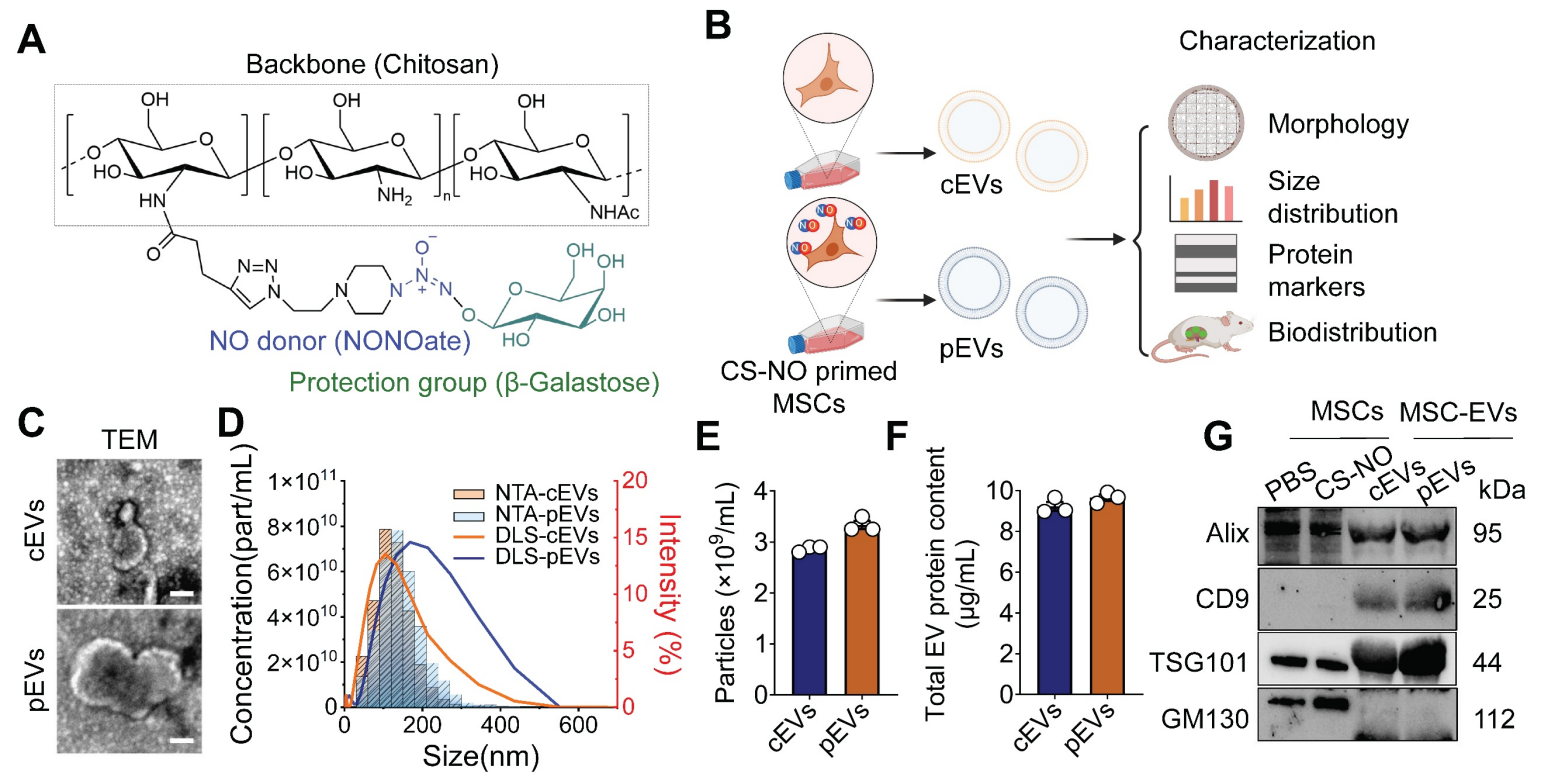
**Generation and characteristics of pEVs.** (A) Chemical structure of the Chitosan - based nitric oxide - releasing biomaterials (CS-NO). (B) Schematic illustration generation and characteristic of MSC-EVs with or without CS-NO priming. Briefly, MSCs were treated with CS-NO. cEVs, EVs from normal MSCs; pEVs, CS-NO-primed MSCs-derived EVs. Created by Biorender. (C) Representative TEM images of cEVs and pEVs. Scale bar, 200 nm. (D) The size distribution of cEVs and pEVs was measured by DLS and NTA. (E-F) Particle concentration (E) and total protein concentration (F) were measured for cEVs and pEVs by NTA (E) and BCA assay (F), respectively. (G) Western blotting analysis of EV markers on MSCs and MSC-EVs. GM130 was used as a negative control. Data are presented as means ± SEM, n = 3; ns, not significant.

CS-NO was synthesized as reported previously (Figure 1A) 18, 20, 21, and a final concentration of 0.025 mg/mL was used to culture MSCs. The NO level in the culture system, detected with the DAF-2 fluorescence probe, was significantly higher in the CS-NO coated group (Figure S1). Subsequently, we isolated and purified MSC-EVs obtained according to previously described protocols 23 to evaluate the characteristics of cEVs and pEVs (Figure 1B). TEM imaging displayed a 'cup-shaped' morphology of cEVs and pEVs (Figure 1C). Subsequently, NTA and DLS showed that the cEVs and pEVs were between 100-300 nm in size (Figure 1D). However, cEVs and pEVs generally exhibited similar particle sizes (Figure 1E) and total protein content (Figure 1F). Using WB analysis, we confirmed the expression of EV markers and showed that both cEVs and pEVs expressed the EV surface marker CD9 and the cytoplasmic protein markers Alix and TSG101, but not GM130; pEVs tended to show slightly higher levels of expression for TSG101 (Figure 1G). These results from TEM, NTA, DLS, and WB indicated that the EVs had been successfully extracted from the MSCs.

Following the identification of MSC-EVs, we evaluated their distribution and assessed whether they could be internalized by injured kidneys. We used noninvasive fluorescence imaging to track the distribution of MSC-EVs at designated time points after intravenous injection of 0.1 mL of 100 μg of AIE-EVs into AKI mice. Significant fluorescence signals were observed in the AKI kidneys of the AIE-EV groups 2 h post-injection, with a peak at 12 h, followed by a gradual intensity decline after 12 h post-injection (Figure S2). We also used the commonly used DiR dye to label EVs and observed a significant accumulation of fluorescent signals in the organs 12 h post-injection (Figure S3).

### pEV showed preferential renal function restoration and renal lesions amelioration in AKI mice

**Figure 2:**
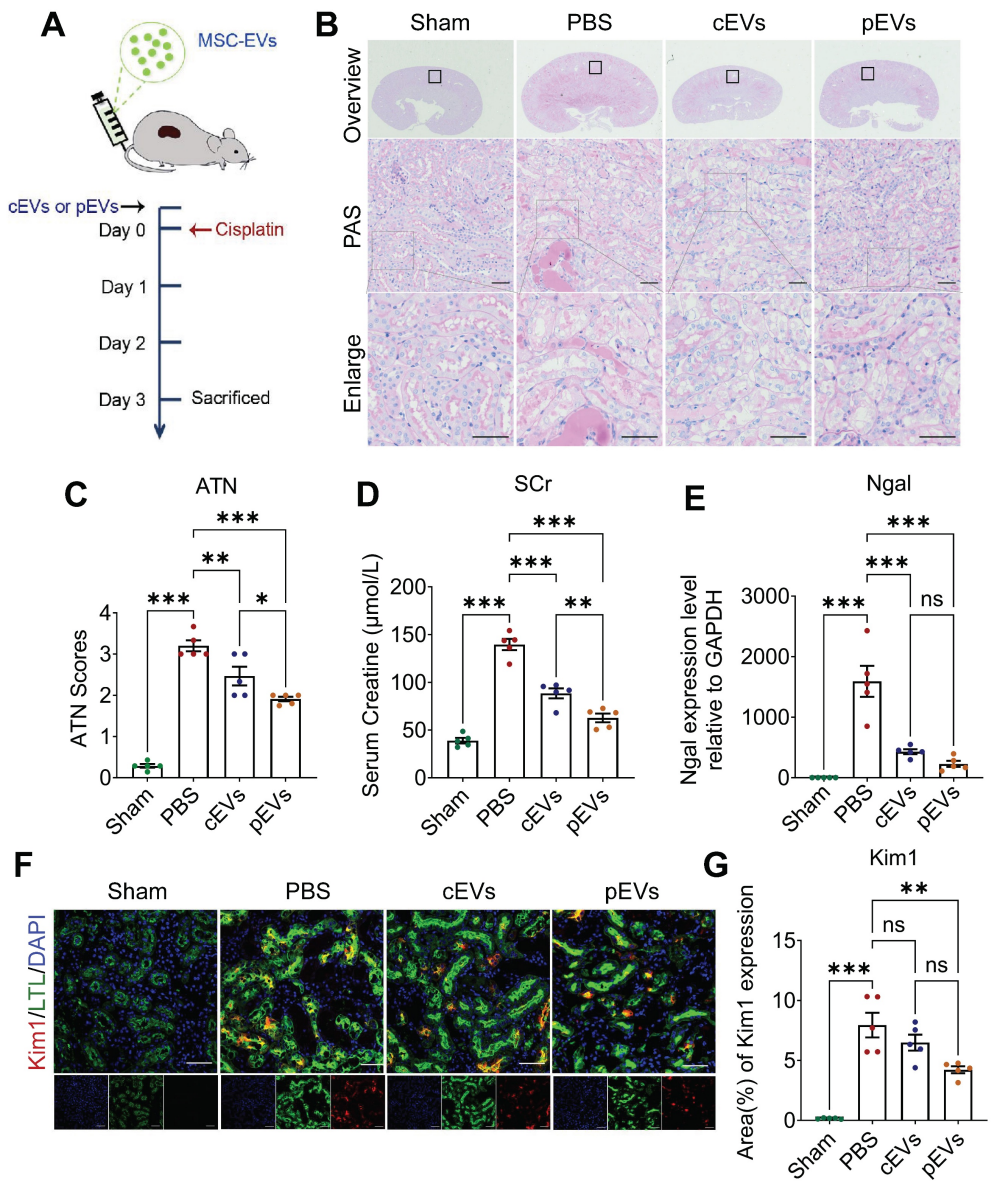
**pEV showed preferential renal function restoration and renal lesions amelioration in AKI mice.** (A) Experimental schedule for the treatment of the AKI mice. Mice using a single 20 mg/kg intraperitoneal injection of cisplatin followed by intravenous injection of PBS, cEVs and pEVs before cisplatin injection. PBS injection served as the negative control. (B) Representative kidney images following PAS staining after the indicated treatments. (C) Quantitative histological assessment of ATN of PAS staining on day 3 after AKI. (D) Renal function parameters, comprising serum creatinine levels, were assessed on day 3 following the onset of AKI. (E) Expression level of kidney injury marker Ngal in the AKI by RT-qPCR. (F) Representative immunofluorescence images showing the expression of kidney injury marker Kim1. Kim1 (red), LTL (green) and DAPI (nuclear; blue). (G) Quantification of Kim1 expression in (F). Data are presented as means ± SEM, n = 5; *p < 0.05, **p < 0.01, ***p < 0.001. ns, not significant. Scale bar, 50 μm.

Given that pre-treatment with CS-NO can enhance the viability of MSCs and augment their paracrine function, we hypothesized that MSC-derived EVs primed with CS-NO enhance the therapeutic efficacy for AKI 28. Based on previous studies, we induced AKI in mice using a single 20 mg/kg intraperitoneal injection of cisplatin 29. Two hours before the cisplatin injection, the mice in each group were injected with PBS, cEVs, or pEVs into the tail vein to determine the protective effect of EVs (Figure 2A). Histopathological damage was assessed using PAS staining (Figure 2B) and ATN scoring (Figure 2C) to evaluate the therapeutic effects. PAS staining demonstrated that the PBS group exhibited noticeable tubular epithelial cell shedding and a high abundance of protein casts compared to the control group. Notably, the cEV and pEV groups showed significant improvements in the aforementioned pathological damage, with a more remarkable effect observed in the cEV group (Figure 2B, C). On day 3 after administration, the mice in the PBS group had higher SCr levels, suggesting significant impairment of renal function (Figure 2D). In contrast, compared to the PBS group, the SCr levels of mice in the cEV and pEV groups were significantly reduced. Remarkably, the pEV group exhibited more significant functional improvement.

To provide additional evidence for the ability of cEVs or pEVs to mitigate renal damage in an AKI model, we assessed the levels of markers of kidney injury, including kidney injury molecule-1 (Kim1) and neutrophil gelatinase-associated lipocalin (Ngal). The measurements of Kim1 and Ngal levels confirmed that pEVs had the best therapeutic efficiency (Figure 2E-G). These findings revealed a more pronounced reduction in Kim1 levels in the kidneys treated with pEVs than in those treated with PBS in the AKI mouse models. Based on these results, both cEVs and pEVs are effective in treating AKI-induced injuries, but pEVs exhibit superior therapeutic efficacy.

### Superior reduction of oxidative stress and cell death in AKI after pEVs treatment

**Figure 3:**
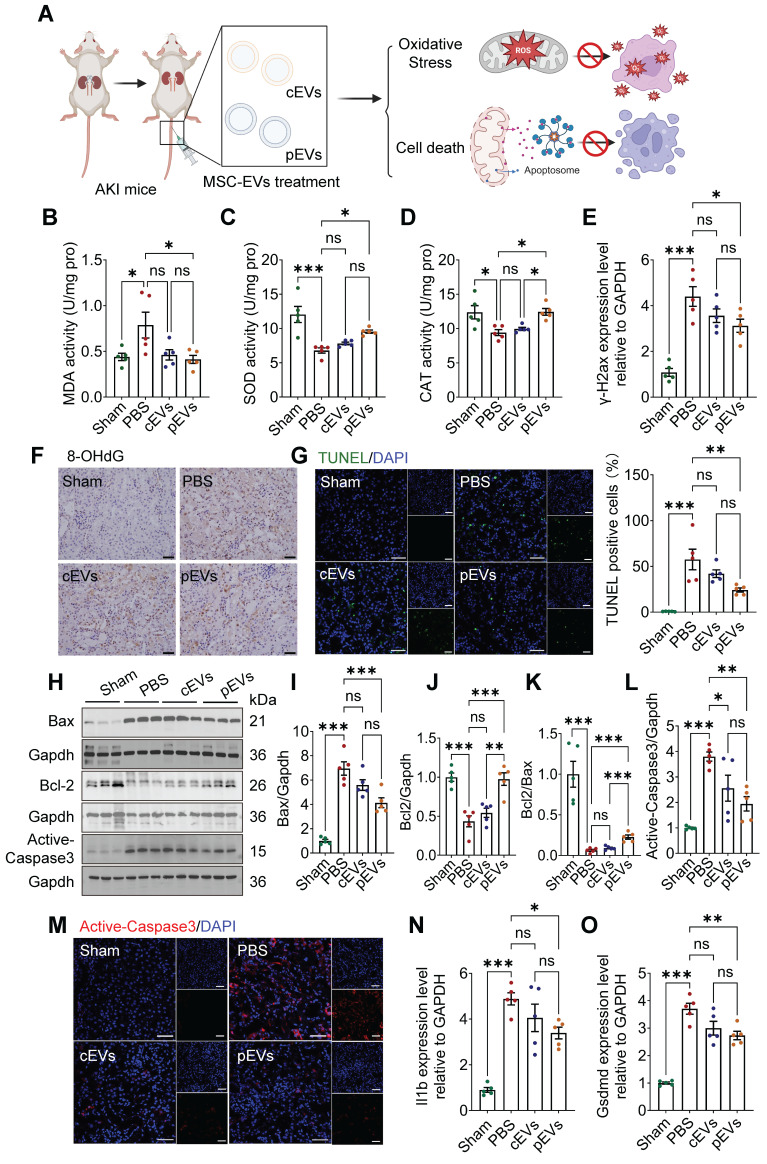
** Superior reduction of oxidative stress and cell death in AKI after pEV treatment.** (A) Schematic diagram illustrates how pEVs to rescue oxidative stress and cell death in AKI. Created by Biorender. (B-D) The levels of lipid peroxidation indicator MDA activity (B), SOD activity (C) and CAT activity (D) of kidney tissues in each group. (E) RT-qPCR analysis of differentially expressed DNA damage marker of γ-H2ax after the indicated treatments. (F) Oxidative DNA damage indicator 8-OHdG expression was detected by IHC staining. Scale bar, 20 μm. (G) Representative immunofluorescence staining and quantification of TUNEL images in kidneys after the indicated treatments. TUNEL (green) and DAPI (nuclear; blue). Scale bar, 50 μm. (H-L) Representative Western blot images (H) and quantitative analyses (I-L) of the apoptosis markers Bax, Bcl2, Bcl2/Bax and active-caspase3. (M) Representative immunofluorescence staining of active-caspase3 images in kidneys after the indicated treatments. Scale bar, 50 μm. (N-O) Pyroptosis markers Il1b (N) and Gsdmd (O) transcript from kidney tissues on day 3 in AKI. Data are presented as means ± SEM, n = 5; *p < 0.05, **p < 0.01, ***p < 0.001. ns, not significant.

Mitochondria support multiple functions in cells, such as maintenance of redox balance, initiation of inflammation and regulation of cell death 30. Increasing evidence has implicated mitochondrial oxidative stress, and cell death has been identified as the main cause of AKI, leading to subsequent extensive tissue damage 31, 32. We tested whether pEV treatment could reduce mitochondrial-related oxidative damage and cell death (Figure 3A). The levels of the peroxidation indicator MDA, as well as SOD and CAT levels in the kidneys, were measured to evaluate oxidative stress status 33.

The results showed a significant increase in MDA levels and a reduction in SOD and CAT activity in the AKI group compared to those in the control group, indicating that the kidney had been subjected to serious oxidative stress (Figure 3B-D). pEVs rescued kidney tissues from oxidative stress by preventing the abnormal elevation of MDA levels and reduction of SOD and CAT activity (Figure 3B-D). Moreover, we examined the levels of phosphorylated histone H2AX (γ-H2AX) and 8-hydroxy-2'-deoxyguanosine (8-OHdG), as markers of oxidative damage to nucleic acids (Figure 3E, F). Our results showed that the levels of γ-H2AX and 8-OHdG increased significantly in the AKI group, while a significant decrease was observed in the pEV treatment group and no difference in the cEV treatment group (Figure 3E, F).

Apoptosis increased significantly as well, which was manifested by apoptotic cell death, as assessed using TUNEL staining (Figure 3G) and the expression of apoptosis-related proteins Bax, Bcl-2, Bcl-2/Bax, and active-caspase 3 using WB analyses and immunofluorescence (Figure 3H-M) in the AKI group. Compared to the AKI group, the apoptosis markers (Bax and active-caspase 3) were visibly reduced in the pEV and cEV treatment groups, and the effect was more significant in the former (Figure 3H, I, L-M). Moreover, anti-apoptosis markers (Bcl-2 and Bcl-2/Bax) were increased in the pEV treatment group (Figure 3J, K). The effects of EVs on pyroptosis were also investigated. The results showed that the expression of Il1b and Gsdmd in the AKI group increased significantly compared with those in the control group, while pEVs lowered the expression of these markers (Figure 3N, O). In conclusion, pEVs rescued oxidative stress and cell death in AKI.

### CS-NO priming enhanced mitochondrial biogenesis within parent cell (MSCs) and their derived pEVs, and subsequently pEVs augmented mitochondria transfer into recipient renal cells

**Figure 4:**
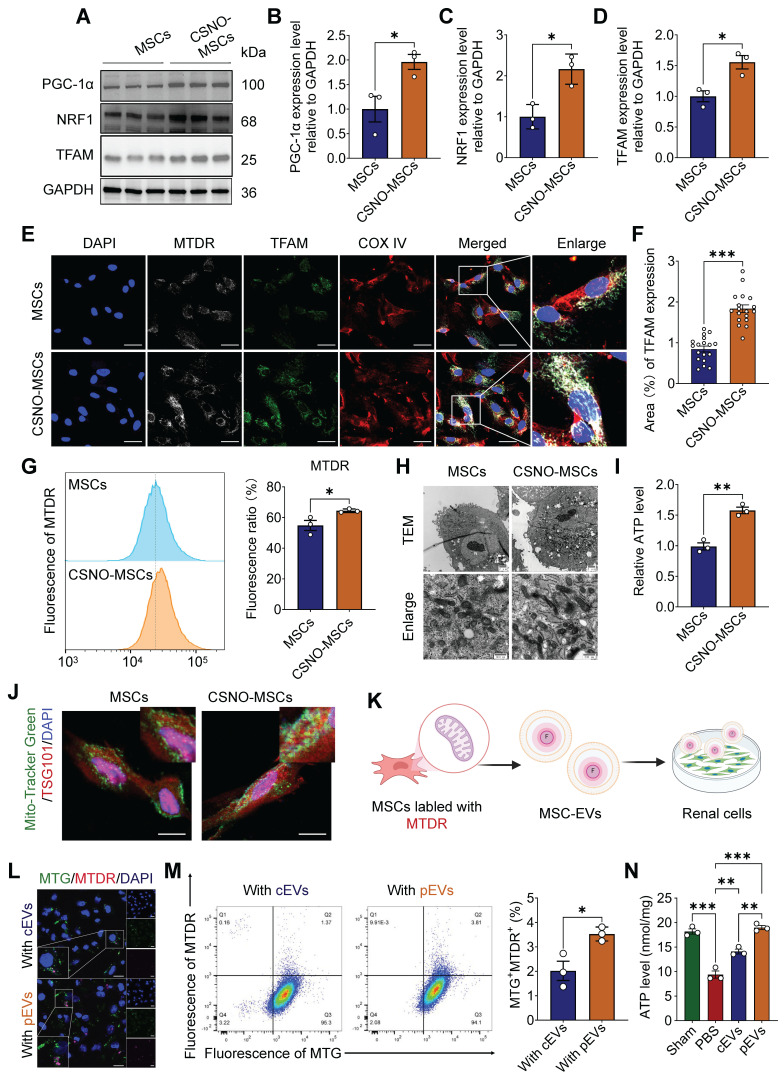
** CS-NO priming enhanced mitochondrial biogenesis within parent cell (MSCs) and their derived pEVs, and subsequently pEVs augmented mitochondria transfer into recipient renal cells.** (A-D) Representative Western blot images (A) and quantitative analyses of mitochondrial biogenesis PGC1-α (B), NRF1 (C) and TFAM (D) in MSCs cultured with or without CS-NO. (E) Representative images of immunofluorescence staining for Mito-tracker deep red (MTDR, gray), the mitochondrial marker COX IV (red) and mitochondrial biogenesis marker TFAM (green) in MSCs with or without CS-NO conditions, showing improved mitochondrial mass and biogenesis of CSNO-MSCs. Scale bar, 20 μm.(F) Quantification of the TFAM fluorescence in (E). (G) Flow cytometry analysis of MTDR and quantification of the fluorescence intensity in MSCs primed with CS-NO, indicating an increase in the mass of mitochondria. (H) Representative TEM of mitochondria in MSCs were cultured with or without CS-NO. Scale bar, 500 nm. (I) Relative intracellular ATP levels of MSCs with or without CS-NO treatment. (J) Represent images of TSG101 (red), together with Mito-tracker green (MTG, green) were detected by immunofluorescence staining within CS-NO treated MSCs, indicating sorting of mitochondrial cargo into pEVs. Scale bar, 20 μm. (K-L) Representative immunofluorescence staining images of mitochondrial content transfer to the mitochondria of HK-2 cells via transport of EVs. MTG fluorescence indicates mitochondria of HK-2 cells, MTDR fluorescence indicates mitochondria of pEVs or cEVs. Scale bar, 50 μm. (M) Flow cytometry detection of the percentage of MTDR^+^MTG^+^ cells among HK-2 cells indicating increasing active mitochondrial content transfer within pEVs. (N) The levels of intracellular ATP in cisplatin-injured HK-2 treated with cEVs and pEVs. Data are presented as means ± SEM, n = 3; *p < 0.05, **p < 0.01, ***p < 0.001. ns, not significant.

We confirmed that CS-NO enhances the renal protective capability of MSC-EVs, but the underlying mechanism remains unclear. Based on previous studies, NO plays a pivotal role in maintaining mitochondrial homeostasis, with several studies indicating that low concentrations of NO can act as a molecular switch, triggering mitochondrial biogenesis and being involved in mitochondrial quality control (MQC) 16, 17. To explore whether CS-NO participates in MQC in MSCs, Peroxisome proliferator-activated receptor gamma coactivator-1α (PGC-1α) and two downstream factors of the mitochondrial biogenesis pathway, nuclear respiratory factor 1 (NRF1) and mitochondrial transcription factor A (TFAM), were evaluated. The expression of PGC-1α, NRF1 and TFAM was significantly upregulated after CS-NO treatment (Figure 4A-D). These results confirmed that CS-NO treatment activated PGC-1α/NRF1/TFAM signaling, thereby inducing mitochondrial biogenesis in MSCs.

MTDR effectively accumulates and emits fluorescence when mitochondria are active and maintain a normal membrane potential 34. We assessed the co-localization of MTDR, COX IV, and the mitochondrial biogenesis marker TFAM. The results revealed complete co-localization of MTDR with TFAM in MSCs independent of CS-NO treatment and that TFAM content increased in cells that also contained increased MTDR (Figure 4E, F). The mean fluorescence intensity of MTDR expression in MSCs was analyzed using flow cytometry and immunofluorescence. The results showed that the expression of MTDR in MSCs tended to increase after CS-NO treatment (Figure 4G). Meanwhile, MSCs primed with CS-NO demonstrated a greater prevalence of mature and elongated mitochondria with denser intra-mitochondrial cristae and a more compact matrix compared to MSCs without CS-NO treatment (Figure 4H). Further analysis identified increased ATP production (Figure 4I) with increased mitochondrial biogenesis. The results showed that mitochondrial biogenesis improved after CS-NO treatment, enhancing the ATP generation capacity.

Intrinsic cellular changes alter the cargo of EVs secreted from native cells 35, and inducing mitochondrial biogenesis enhances the capacity of MSCs for intercellular mitochondrial transfer 12. Recent studies confirmed that enhanced mitochondrial biogenesis increases the secretion of mitochondria-containing EVs 12, 36. To confirm that the enhanced transfer capacity of active mitochondrial contents via pEVs, the colocalization of TSG101 with MTDR was significantly increased by CS-NO treatment (Figure 4J), indicating sorting of active mitochondrial cargo into EVs.

Subsequently, to further confirm that active mitochondrial contents are transferred to damaged renal cells via pEVs, 8 h after cEV or pEV treatment, exogenous active mitochondrial contents with Mito-Tracker staining from EVs (MTDR) were found to co-localize with mitochondria from HK-2 cells (MTG) after cisplatin treatment (Figure 4K and Figure S4). The results highlight that, compared with cEVs, pEVs mediated transfer of active mitochondrial contents significantly increased in the mitochondria of cisplatin-treated HK-2 cells, and co-localization with recipient cell mitochondria (MTG) was significantly enhanced (Figure 4L, M and Figure S5). Functional assays demonstrated that both cEVs and pEVs effectively restored ATP depletion in cisplatin-induced HK-2 cells, with pEVs exhibiting superior restorative efficacy (Figure 4N).

### pEV delivered mitochondrial contents to renal tubular cells in restoring renal bioenergetic capacity

**Figure 5:**
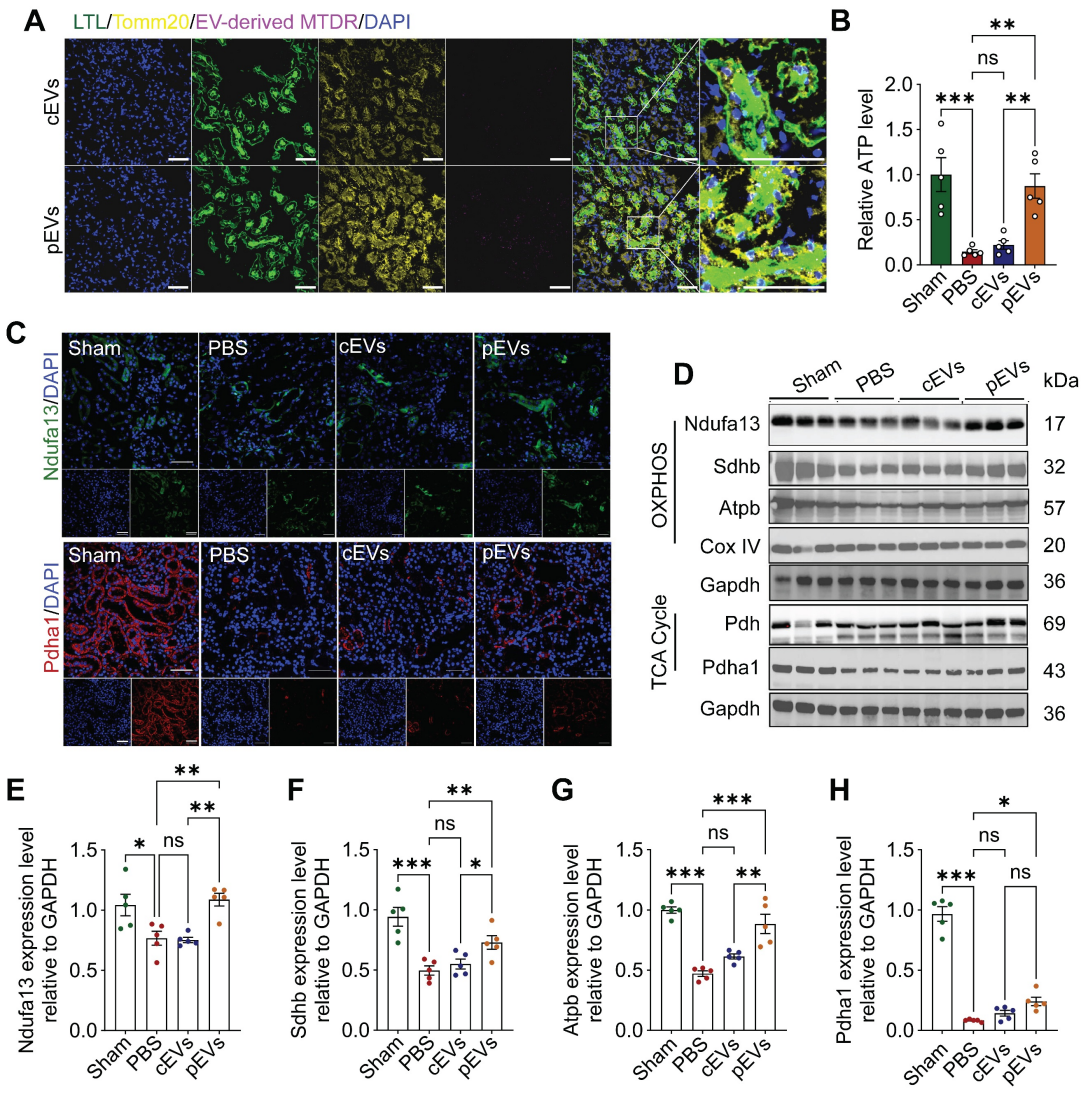
** pEV delivered mitochondrial contents to renal tubular cells in restoring renal bioenergetic capacity.** (A) Representative immunofluorescence images of kidney sections demonstrate EV-mediated mitochondrial contents delivery to renal cells in AKI mice. Co-localization of fluorescently labeled renal tubule (LTL; green), mitochondria of RTECs (Tomm20; yellow), and pEV-associated mitochondrial content were labeled with MTDR (purple). Scale bar, 50 μm. (B) Relative intracellular ATP levels in AKI mice. (C) Representative immunofluorescence images of mitochondrial protein content Ndufa13 and Pdha1 in the kidneys. Scale bar, 50 μm. (D-H) Representative Western blot images (D) and quantitative analyses (E-H) of the mitochondrial OXPHOS markers Ndufa13 (E), Sdhb (F), Atpb (G), Cox IV and TCA cycle markers Pdh, Pdha1 (H), showing the changes of mitochondrial metabolism after cEV or pEV treatment in AKI. Data are presented as means ± SEM, n = 5; *p < 0.05, **p < 0.01, ***p < 0.001. ns, not significant.

Notably, active mitochondria delivered by pEVs were detected in the kidney tissue through immunofluorescence staining. The results revealed that the labeled mitochondria were successfully transferred to renal cells. Mitochondria in the kidney (Figure 5A) were also affected. Moreover, we found that ATP levels decreased in the AKI group, and this effect was reversed after pEV treatment (Figure 5B). These findings were consistent with our in vitro results. Next, we analyzed mitochondrial metabolism by assessing the levels of mitochondria-associated proteins (Ndufa13, Atpb, Sdhb, and Pdha1). The results showed significant decreases in the expression of mitochondrial proteins in the AKI group, and pEV treatment was more efficient in rescuing the decreased Ndufa13, Sdhb, Atpb, and Pdha1 protein levels (Figure 5C-H). Together, these results demonstrate the potential of pEVs to facilitate the transfer of active mitochondrial contents into the mitochondria of renal tubular epithelial cells (RTECs) and more effectively rescue mitochondrial energetics in kidneys for AKI treatment.

### pEVs delivered mitochondrial contents in recipient cells, recovering renal mitochondrial homeostasis

**Figure 6:**
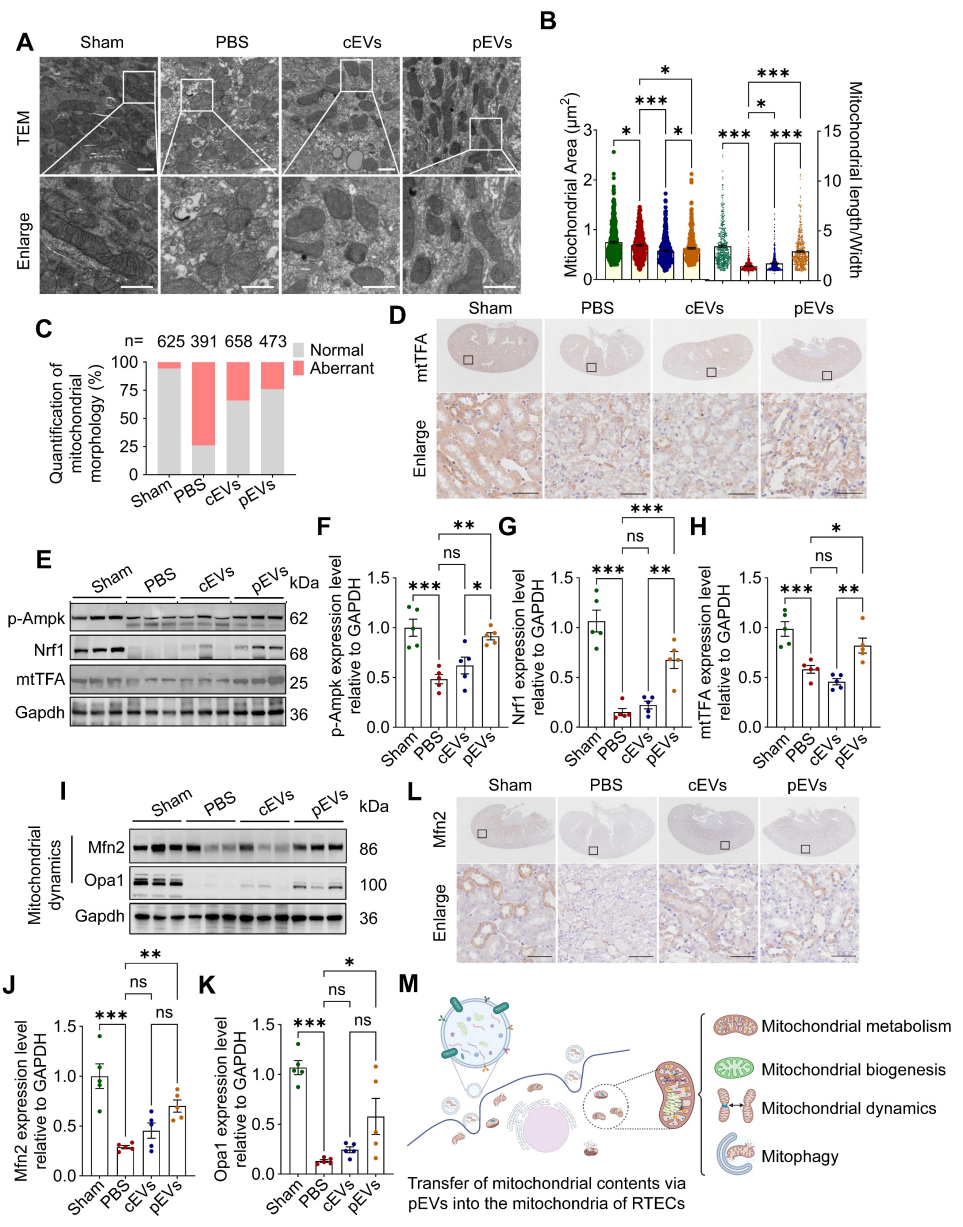
** pEVs delivered mitochondrial contents in recipient cells, recovering renal mitochondrial homeostasis.** (A) Representative TEM of kidneys, indicating mitochondrial damage (swollen, fragmented and dysmorphic) in the kidney tissues of mice with AKI, and the shapes and architecture of mitochondria are preserved in the group treated with pEVs. Scale bar, 500 nm. (B) Quantitative analysis of mitochondrial area (left) and length/width (right) in kidney tissues. (C) Quantitative analysis of the number of normal or aberrant mitochondria in kidney tissues (n, number of mitochondria analyzed). (D) Immunohistochemical staining of mitochondrial biogenesis marker mtTFA in the kidney from AKI mice. Scale bar, 50 μm. (E-H) Representative Western blot images (E) and quantitative analyses (F-H) of the mitochondrial biogenesis markers p-Ampk (F), Nrf1 (G), mtTFA (H). (I-K) Representative Western blot images (I) and quantitative analyses (J-K) of the mitochondrial fusion proteins Mfn2 (J) and Opa1 (K). (L) Immunohistochemical staining of mitochondrial fusion protein Mfn2 in the kidney from AKI mice. Scale bar, 50 μm. (M) The schematic diagram illustrates the assessment of EV-mediated transfer of mitochondrial contents into the mitochondria of RTECs for facilitating renal mitochondrial homeostasis restoration in AKI. Created by Biorender. Data are presented as means ± SEM, n = 5; *p < 0.05, **p < 0.01, ***p < 0.001. ns, not significant.

Our study demonstrates that CS-NO stimulation of MSCs enhances the transfer of active mitochondrial contents via pEVs. The transfer of mitochondrial contents can lead to alterations in the functional profile of the mitochondria within recipient cells 37. To confirm the role of pEVs in facilitating the transfer of mitochondrial contents and in restoring renal mitochondrial homeostasis, we conducted quantitative assessments of mitochondrial morphology, function, biogenesis, and dynamics in AKI mouse model. TEM images from the AKI group revealed that the mitochondria were damaged, exhibiting a swollen or spherical shape, hazy structure, and disrupted cristae (Figure 6A). There was a significant decrease in the mitochondrial area and length/width ratio (Figure 6B), along with an increased ratio of aberrant mitochondria (Figure 6C). Compared with the AKI group, cEV and pEV treatment recovered mitochondrial morphology, with pEVs demonstrating a more pronounced protective effect (Figure 6A-C).

The MQC system can accurately maintain the dynamic balance of mitochondria 38. This system mainly involves three distinct mechanisms, namely mitochondrial biogenesis, mitochondrial dynamics (fusion and fission), and mitophagy 7, 39. We observed that the level of phosphorylated adenosine 5'-monophosphate-activated protein kinase (p-Ampk) protein decreased significantly in the AKI and cEV groups compared to that in the control group, and that pEV treatment attenuated this decrease (Figure 6E, F). Ampk acts as a master regulator for mitochondrial biogenesis by trans- activating Nrf1 and Tfam, whose levels were significantly decreased in the AKI group compared to those in the control group. pEVs attenuated the decrease in Nrf1 and Tfam protein expression, whereas cEVs did not restore their expression (Figure 6D, E-H).

Mitochondrial fission/fusion is a critical and dynamic process that maintains functional mitochondria 2. WB and IHC analyses demonstrated that the expression levels of Mfn2 and Opa1 were significantly lower in the AKI group than in the control group, whereas pEV treatment significantly upregulated their protein levels (Figure 6I-L). However, an elevation in Drp1 levels was observed in the pEV group compared to that in the PBS group, with no significant differences noted among the other groups (Figure S6). To explore the effect of pEVs on mitophagy, we assessed the expression levels of Pink1 and Parkin and found that they decreased in the AKI group compared to those in the control group, which was reversed after pEV treatment (Figure S7A-C). The ability of pEVs to recover impaired mitophagy was confirmed by determining the expression of the autophagy markers LC3B II and P62 (Figure S7A, D-F). Collectively, these results reveal that the efficient transfer of active mitochondrial contents via pEVs into the mitochondria of RTECs restores impaired mitochondrial morphology and function by recovering MQC (Figure 6M).

### pEVs were identified to be enriched with functional mitochondrial cargo, whereas mitochondria-depleted pEVs abolished these therapeutic effects

**Figure 7:**
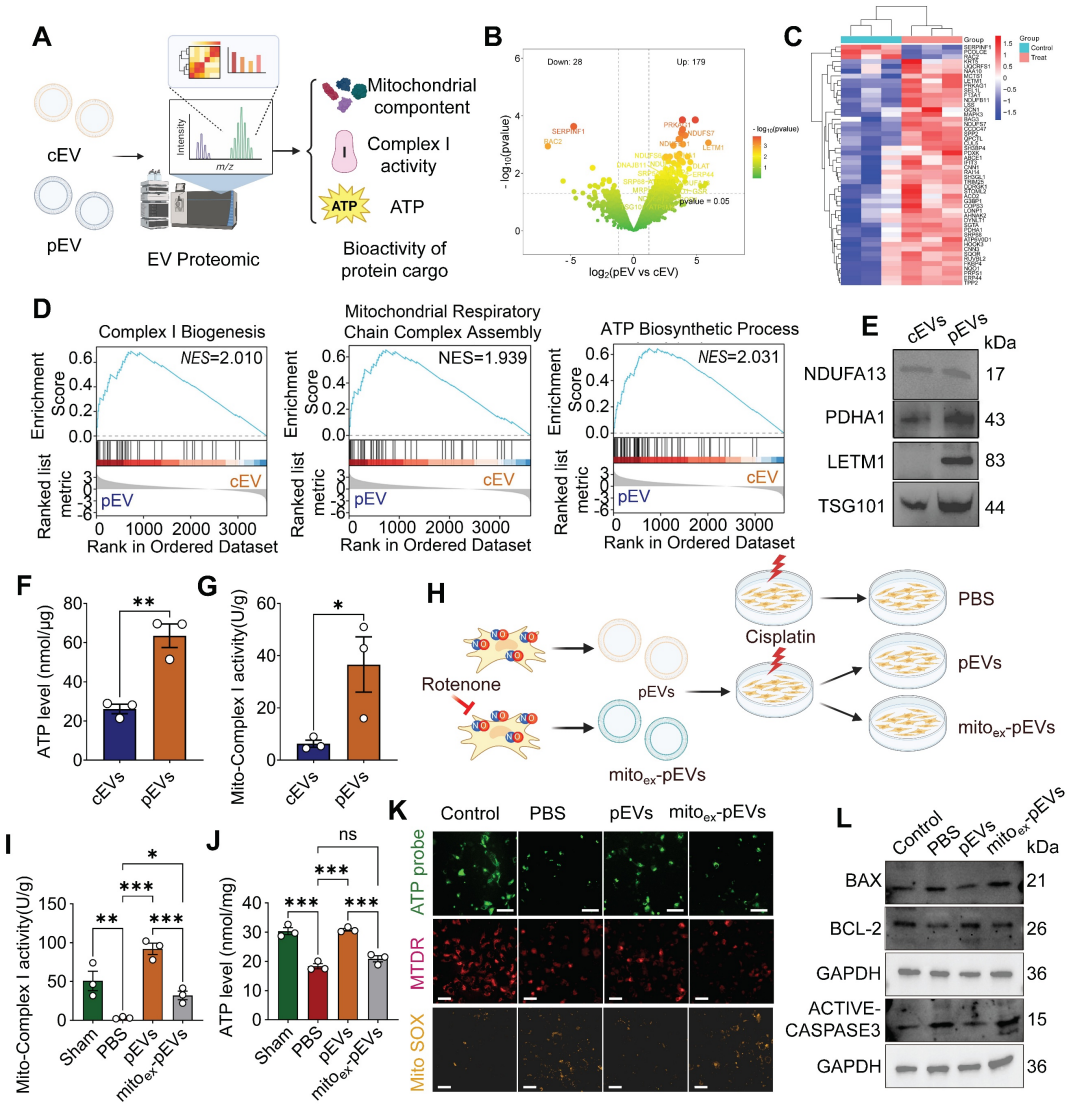
** pEVs were identified to be enriched with functional mitochondrial cargo, whereas mitochondria-depleted pEVs abolished these therapeutic effects.** (A) Illustration suggesting that proteomics analysis increased the encapsulation of mitochondrial components within pEVs and functional validation. Created by Biorender. (B) Volcano map of DEPs between cEVs and pEVs. Red represents up- and down- regulated proteins, and green represents proteins with no significant difference. (C) Heatmap depicting the cluster analysis of the top 50 DEPs in pEVs compared to cEVs. (D) The GSEA of EV cargos revealed enrichment for the mitochondrial respiratory chain complex assembly, citrate cycle TCA cycle and ATP biosynthetic process, is involved in mitochondrial metabolism. (E) Representative western blot images of pEV cargo showing the increased mitochondrial protein content NDUFA13, PDHA1, LETM1 and TSG101. (F-G) The levels of intracellular ATP (F) and mito-complex I activity (G) in cEVs and pEVs. (H) Schematic diagram of the experimental groups set up for comparison with the groups Control (without cisplatin), PBS, pEVs, and mito_ex_-pEVs. mito_ex_-pEVs were isolated from NO primed MSCs following pharmacological inhibition of mitochondrial complex activity with rotenone. Created by Biorender. (I-J) Mito-complex I activity (I) and intracellular ATP level (J) in cisplatin-injured HK-2 treated with pEVs and mito_ex_-pEVs. (K) Representative images of ATP level (ATP probe; green; Scale bar, 100 μm), mitochondrial membrane potential (MTDR; red; Scale bar, 50 μm) and ROS expression (mitochondrion-specific probe Mito SOX red; yellow; Scale bar, 50 μm). (L) Representative western blot images of the apoptosis markers Bax, Bcl2 and active-caspase3 in cisplatin-injured HK-2 treated with pEVs and mito_ex_-pEVs. Data are presented as means ± SEM, n = 3; *p < 0.05, **p < 0.01, ***p < 0.001. ns, not significant.

To comprehensively analyze the biological effects of CS-NO on MSC-derived EVs, we performed proteomic analysis and assessed the bioactivity of cEVs and pEVs (Figure 7A). Principal component analysis (PCA) suggested pronounced differences in the protein profiles between cEVs and pEVs (Figure S8A). In total, 207 proteins were differentially expressed between the cEV and pEV groups, including 179 with upregulated and 28 with downregulated expression (Figure 7B, C). Notably, GSEA of the EV cargo revealed enrichment for the complex I biogenesis, mitochondrial respiratory chain complex assembly and ATP biosynthetic process (Figure 7D), indicating that the contents of pEVs are involved in mitochondrial metabolism and function. Consistent with the aforementioned findings, KEGG and GO analyses showed that DEPs were enriched in the ATP metabolic process, electron transport chain, mitochondrial matrix, and electron transport chain activity (Figure S8B). This key protein expression was further confirmed through WB, which showed increased mitochondrial protein contents (NDUFA13, PDHA1, LETM1) and intramembrane marker TSG101 in pEVs (Figure 7E). The findings described above demonstrate that pEVs have increased mitochondrial protein contents. We measured the mitochondrial bioactivity of pEVs and found that they exhibited stronger ATP production and mitochondrial respiratory chain Complex I activity compared to cEVs (Figure 7F-G). These results confirm that pEVs have enhanced mitochondrial bioactivity, likely due to their higher content of mitochondrial components.

To elucidate the functional contribution of mitochondrial components in pEVs, we pharmacologically inhibited mitochondrial respiratory chain complex activity by rotenone in NO-primed MSCs and assessed subsequent mitochondrial recovery (Figure 7H). The results revealed that PBS group exhibited significant reductions in complex I activity and ATP production, whereas pEVs administration restored these parameters to near-basal levels (Figure 7I-J). Strikingly, mitochondrial depletion of pEVs (mito_ex_-pEVs) abolished these restorative effects. Consistently, fluorescence imaging demonstrated diminished ATP intensity and mitochondrial mass (Mito-tracker) alongside elevated mitochondrial ROS (Mito SOX) in PBS group, which were robustly reversed by pEV treatment (Figure 7K). Conversely, mito_ex_-pEVs failed to mitigate these deficits. Furthermore, pEVs attenuated cisplatin-induced apoptosis by downregulating pro-apoptotic markers Bax and active-caspase-3 while upregulating Bcl-2, effects that were nullified by mito_ex_-pEVs (Figure 7L).

## Discussion

The novel findings of this study are as follows: 1) NO enhances mitochondrial biogenesis; 2) pEVs contain a higher abundance of mitochondrial functional proteins than cEVs; 3) EV-mediated transfer of active mitochondrial cargo restores mitochondrial homeostasis in AKI; and 4) intravenous injection of pEVs reduces oxidative stress and cell death in AKI, thereby improving renal function.

Numerous studies have demonstrated the potential of MSC-EVs for the treatment of various diseases, particularly in renal repair, including AKI 40-44. However, the practical use of MSC-EVs is limited by their low yield and functionality 45. Previous studies have attempted to enhance the therapeutic effects of EVs by activating and engineering parental cells, loading drugs into EVs, and modifying the surface of EVs 14, 46. Recent studies have explored the use of NO-releasing biomaterials to enhance the therapeutic potential of MSCs. As an important signaling molecule, NO plays a critical role in regulating the survival, migration, differentiation, and paracrine behavior of stem cells 28. Combining MSCs with NO-releasing biomaterials has been demonstrated to enhance their angiogenic, anti-apoptotic, and antioxidative stress capabilities, with great potential for treating peripheral ischemia, kidney diseases, and cardiovascular disease 18, 31, 47, 48. In this study, we pretreated MSCs with an NO-releasing polymer, which was linked to chondroitin sulfate and was capable of maintaining a stable low-concentration (nM-level) release for at least 48 h 18, 20, 21. Chronic, modest increases in NO levels have been shown to offer cellular protection and exert effects against oxidative stress in various cell types. Notably, we found that EVs derived from NO-pretreated MSCs exhibited a superior ability to promote renal repair, highlighting the potential of this approach to enhance the therapeutic efficacy of MSC-EVs in treating kidney injury.

Previous research has demonstrated that treatment with low concentrations of NO can preserve the pluripotency of stem cells and induce mitochondrial biogenesis, primarily through the activation of cyclic guanosine monophosphate mediated by guanylate cyclase 49. We conducted a series of experiments to demonstrate that NO-releasing biomaterials drive mitochondrial biogenesis in MSCs. EVs are emerging as important vehicles for the transport of regulatory proteins and functional miRNAs, with certain subgroups of EVs enriched with different mitochondrial contents that can carry mitochondrial contents and affect the metabolic state of target cells 50. Proteomic analysis revealed that compared to cEVs, pEVs contained more mitochondrial respiratory chain proteins. Enhancing mitochondrial biogenesis in donor cells can increase the mitochondrial load in EVs. Dave et al. demonstrated that stimulating intrahepatic biliary epithelial cells with resveratrol, a PGC-1α activator, resulted in a significant increase in both the mitochondrial content of EVs and the total number of mitochondria-containing EVs 51. Large EVs are also enriched in metabolic enzymes and mitochondrial proteins 52. EV size heterogeneity reflects distinct biogenesis pathways and cargo composition 53. Stressors like NO preconditioning may enhance ESCRT-mediated EV biogenesis, favoring larger vesicles capable of packaging metabolic regulators 54. Notably, hypoxia or exercise-induced EVs similarly exhibit increased size correlated with metabolic reprogramming and angiogenesis 55-57. Thus, pEVs' larger size likely enhances therapeutic efficacy by enabling cargo enrichment (e.g., mitochondrial proteins), improved stability, and intercellular communication.

In our study, NO priming induces mitochondrial biogenesis and upregulates TSG101 on mitochondrial membranes. This process may serve two synergistic purposes: First, TSG101 enrichment promotes mitophagy by enhancing Parkin recruitment 58, ensuring the transfer of functional mitochondria in MSCs. Second, TSG101 acts as a cargo-sorting hub to direct mitochondrial components (e.g., mtDNA, mitochondrial component, and metabolites) into EVs 59. Notably, PINK1--a key mitophagy regulator--has been shown to cooperate with TSG101 in a kinase-independent manner to package mitochondrial components into EVs 60, which aligns with our observation of increased TSG101 in pEVs. TSG101-enriched pEVs likely exert therapeutic effects via two pathways: (1) Delivery of functional mitochondrial components: Transferring mitochondrial components to injured renal tubular epithelial cells rescues mitochondrial bioenergetics and mitigates oxidative damage; (2) Activation of endogenous repair pathways: TSG101 may directly interact with recipient cell machinery (e.g., Parkin or ESCRT components) to amplify mitophagy and suppress inflammasome activation--a critical step in alleviating renal ischemia-reperfusion injury. The metabolic remodeling induced by NO priming may further enhance pEV-mediated mitochondrial "metabolic rescue" effects, explaining the superior efficacy of TSG101-high EVs in promoting renal functional recovery. Therefore, inducing mitochondrial biogenesis though PGC-1α/NRF1/TFAM in MSCs after CS-NO treatment significantly contributes to improving the efficiency of mitochondria transfer.

The amount of mitochondrial cargo and pathways for transfer of mitochondrial contents determine the transfer efficacy between cells 12, 61. Previous research has suggested that EV proteins contain necessary components of the tricarboxylic acid cycle and membrane contents involved in energy formation, which may have the potential to reconstruct functional mitochondria 62. Supporting this notion, Ikeda et al. showed that human MSC-EVs mediate mitochondrial transfer, which enhances mitochondrial function by fusing with the mitochondrial network in mouse cells 63. Our study further suggested that compared to cEVs, pEVs carrying mitochondrial protein cargo can better promote the restoration of mitochondrial homeostasis in injured kidneys. Exogenous MSC-EVs can restore respiratory ability in cells with mitochondrial dysfunction in vivo, and these EVs exhibit an independent aerobic respiratory capacity that may be related to their ability to rescue cellular biological functions independent of the mitochondria 64. Our study provides evidence that pEVs can serve as an effective therapeutic strategy to restore mitochondrial function in AKI, thus offering a promising approach for the treatment of this devastating disease.

Our current study has several limitations. NO's bioactivity exhibits a concentration-dependent dual effect: low concentrations of NO (nM level) mediate cell-protective signaling with anti-apoptotic effects, while high concentrations (μM level) can cause DNA damage and mitochondrial dysfunction 65. Therefore, due to dose limitations, NO's narrow therapeutic window partially limits its clinical utility. In this study, our controlled-release NO bioactive material ensures that NO release concentration remains at the nM level, which is within the physiologically safe range.

However, despite recent advances, the clinical application of NO donors remains limited by poor solubility, burst release effects, and off-target nitrosative stress 28. Although our controlled-release NO system partially alleviates some of these issues, scale-up production still faces challenges, including batch variations in NO loading efficiency and the complexity of synthesizing biodegradable materials 31, 66. Moreover, standardizing the use of NO-primed MSCs remains a key challenge. Variations in NO release concentration, exposure time, and treatment methods may affect the quality and functionality of MSC-EVs. Additionally, other challenges to address for clinical translation include large-scale production, storage stability, and the efficient, targeted delivery of EVs. Furthermore, the long-term safety and potential immunogenicity of MSC-EVs must be rigorously evaluated in clinical trials.

## Conclusion

Our findings suggest that EVs play a pivotal role in promoting tissue repair following AKI by restoring renal mitochondrial homeostasis. This effect depends primarily on the effective transfer of active mitochondrial contents mediated by pEVs. Furthermore, the results of our study indicate that the efficient transfer of mitochondrial contents through pEVs can be utilized as a novel nanotherapeutic system for treating kidney diseases. Treating MSCs with a CS hydrogel incorporating controlled release of NO effectively activated mitochondrial biogenesis in MSCs, primarily through the PGC-1α/NRF1/TFAM pathway. The efficient delivery of active mitochondrial contents mediated by pEVs can more effectively restore mitochondrial homeostasis in RTECs. Therefore, MSC-EVs pre-treated with NO-controlled-release hydrogels offer a promising strategy for stem cell-derived product therapy for kidney diseases.

## Supplementary Material

Supplementary figures and table.

## Abbreviations

AKI: Acute kidney injury
MSC-EVs: Mesenchymal stem cell-derived extracellular vesicles
NO: Nitric oxide
ATP: Adenosine triphosphate
EVs: Extracellular vesicles
MSCs: Mesenchymal stem cells
PBS: Phosphate-buffered saline
CS-NO: Chitosan- based nitric oxide - releasing biomaterials
DMEM/F12: Dulbecco's modified Eagle medium/nutrient mixture F-12
CCK-8: Cell Counting Kit-8
TEM: Transmission electron microscopy
DLS: dynamic light scattering
NTA: Nanoparticle tracking analysis
AIE: Aggregation-Induced Emission
BCA: Bicinchoninic acid
PAS: Periodic acid-Schiff
ATN: Acute tubular necrosis
TUNEL: Terminal deoxynucleotidyl transferase-mediated dUTP nick-end labeling
BUN: Blood urea nitrogen
SCr: Serum creatinine
RT-qPCR: Reverse transcription-quantitative polymerase chain reaction
IF: Immunofluorescence
IHC: Immunohistochemical
MDA: Malondialdehyde
SOD: Superoxide Dismutase
CAT: Catalase
MTDR: Mito-Tracker Deep Red FM
MTG: Mito-Ttracker Green
DEPs: Differentially expressed proteins
MSigDB: Molecular Signatures Database
GSEA: Gene Set Enrichment Analysis
ROS: Reactive oxygen species
ANOVA: One-way analysis of variance
Kim1: Kidney injury molecule-1
Ngal: Neutrophil gelatinase-associated lipocalin
γ-H2AX: Phosphorylated histone H2AX
8-OHdG: 8-hydroxy-2'-deoxyguanosine
MQC: Mitochondrial quality control
PGC-1α: Peroxisome proliferator-activated receptor gamma coactivator-1α
NRF1: Nuclear respiratory factor 1
TFAM: Mitochondrial transcription factor A
RTECs: Renal tubular epithelial cells
p-Ampk: Phosphorylated adenosine 5'-monophosphate-activated protein kinase
PCA: Principal component analysis
