# Supplementary material for: Nitric oxide-primed engineered extracellular vesicles restore bioenergetics in acute kidney injury via mitochondrial transfer
Source: Theranostics. 2025 Apr 13;15(11):5499–517. doi: 10.7150/thno.113741 (PMC12036870; doi:10.7150/thno.113741)
Supplement: Supplementary file 1 — Supplementary figures and table. [file thnov15p5499s1.pdf]

Supporting information

Nitric oxide-primed engineered extracellular vesicles restore bioenergetics in acute kidney injury via mitochondrial transfer

Fei Peng[1, 2, #], Xiaoniao Chen[3, #], Lingling Wu[2], Jiayi He[4], Zongjin Li[1], Quan Hong[2], Qiang Zhao[5], Meng Qian[5], Xu Wang[2], Wanjun Shen[2], Tingting Qi[1, 2], Yiyu Huang[2], Guangyan Cai[2, *], Chuyue Zhang[2, 6, *], Xiangmei Chen[1, 2, *]

[1] School of Medicine, Nankai University, Tianjin 300071, China

[2]Department of Nephrology, First Medical Center of Chinese PLA General Hospital, National Key Laboratory of Kidney Diseases, National Clinical Research Center for Kidney Diseases, Beijing Key Laboratory of Kidney Diseases Research, Beijing 100853, China

[3]Department of Ophthalmology, Third Medical Center of Chinese PLA General Hospital, Beijing 100853, China

[4]Clinical Research Center, First Affiliated Hospital of Shantou University Medical College, Shantou, Guangdong 515041, China

[5]Key Laboratory of Bioactive Materials, Ministry of Education, College of Life Sciences, Nankai University, Tianjin, 300071, China.

[6]Kidney Research Institute, Division of Nephrology, West China Hospital, Sichuan University, Chengdu, 610041, China

# These authors contributed equally to the manuscript.

* Corresponding authors.

E-mail addresses: chenxiangmei@301hospital.com.cn and xmchen301@126.com (Xiangmei Chen), zhangcycywch@wchscu.edu.cn (Chuyue Zhang), caiguangyan@sina.com (Guangyan Cai).

Supporting Information Contents:

Figures S1-S8

Table S1

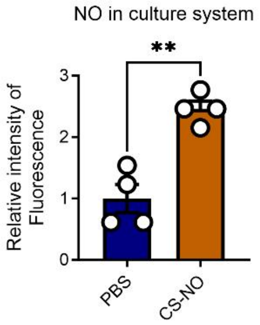

Figure S1. Relative quantification of the level of NO in the culture system determined using the DAF-2 fluorescence probe. Data are presented as means ± SEM. Statistical significance was analyzed using ordinary one-way ANOVA. n = 4; *p < 0.01.

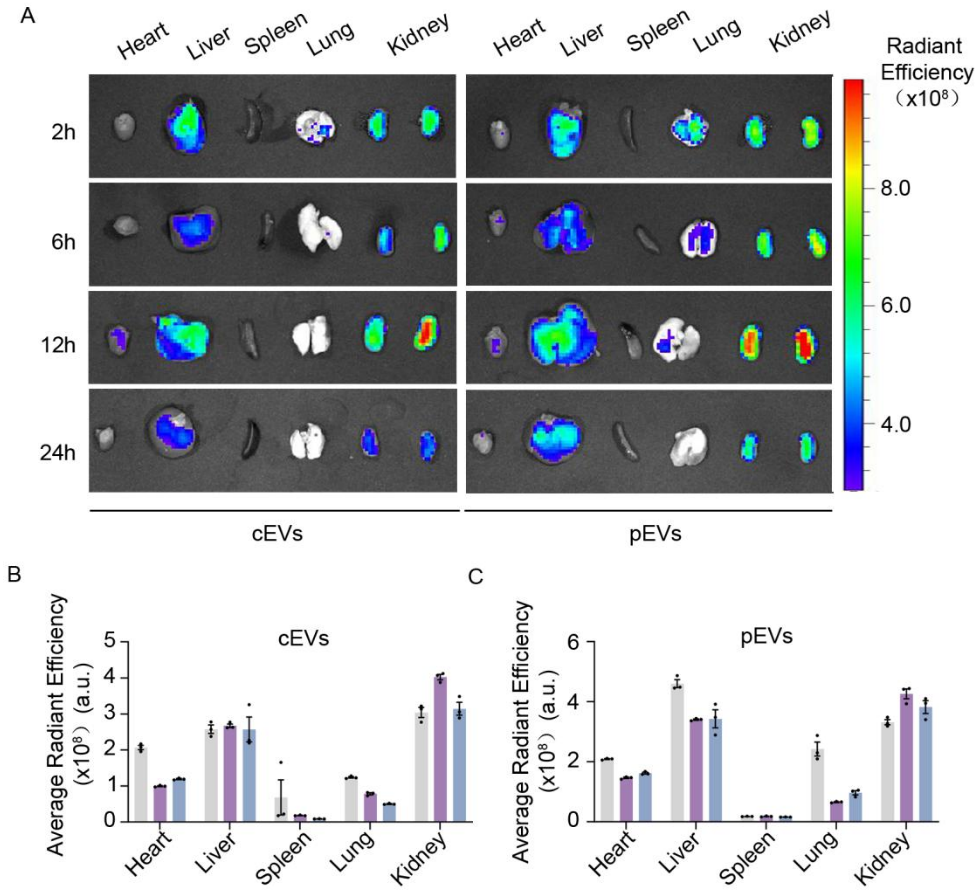

Figure S2. Ex vivo imaging of MSC-EVs in AKI mice. (A) Ex vivo fluorescence biodistribution of vital organs in AIE-EV groups at 2, 6, 12 and 24 h postinjection. (B) Average radiant efficiency changes of vital organs in cEV and pEV groups at selected time points 2 h (gray), 12 h (purple), 24 h (blue). n = 3.

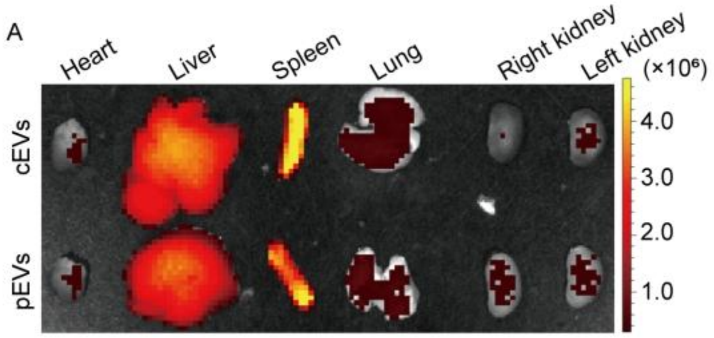

Fig. S3. Real-time imaging of MSC-EVs using DiR tracker. (A) Fluorescence intensity changes of vital organs in DiR-EV groups at 12 h postinjection. n = 3.

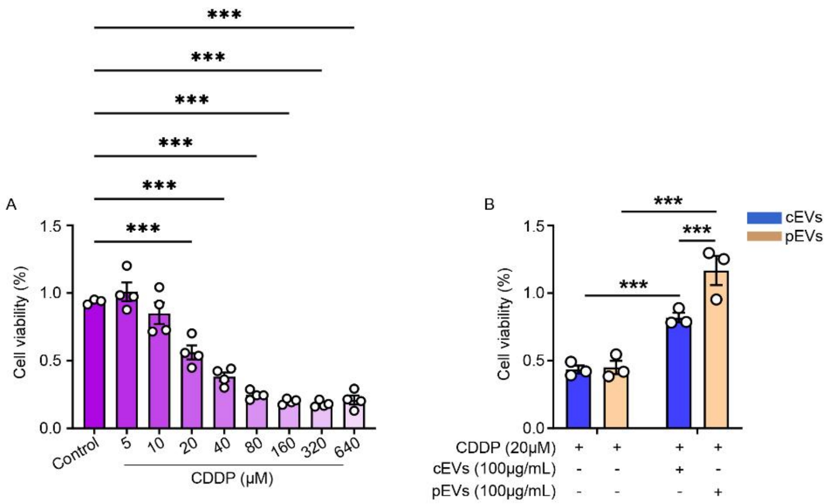

Figure S4. cEVs or pEVs protects HK-2 cells against cisplatin-induced cytotoxicity. (A) Cell viability of HK-2 cells stimulated with the designated doses of cisplatin for 24 h using CCK-8 assay. (B) Cell viability of HK-2 cells after the pretreatment with cEVs or pEVs for 8 h and co-treated with 20 μM cisplatin for 24 h. Data are presented as means ± SEM. Statistical significance was analyzed using ordinary one-way ANOVA. ***$p < 0.001$.

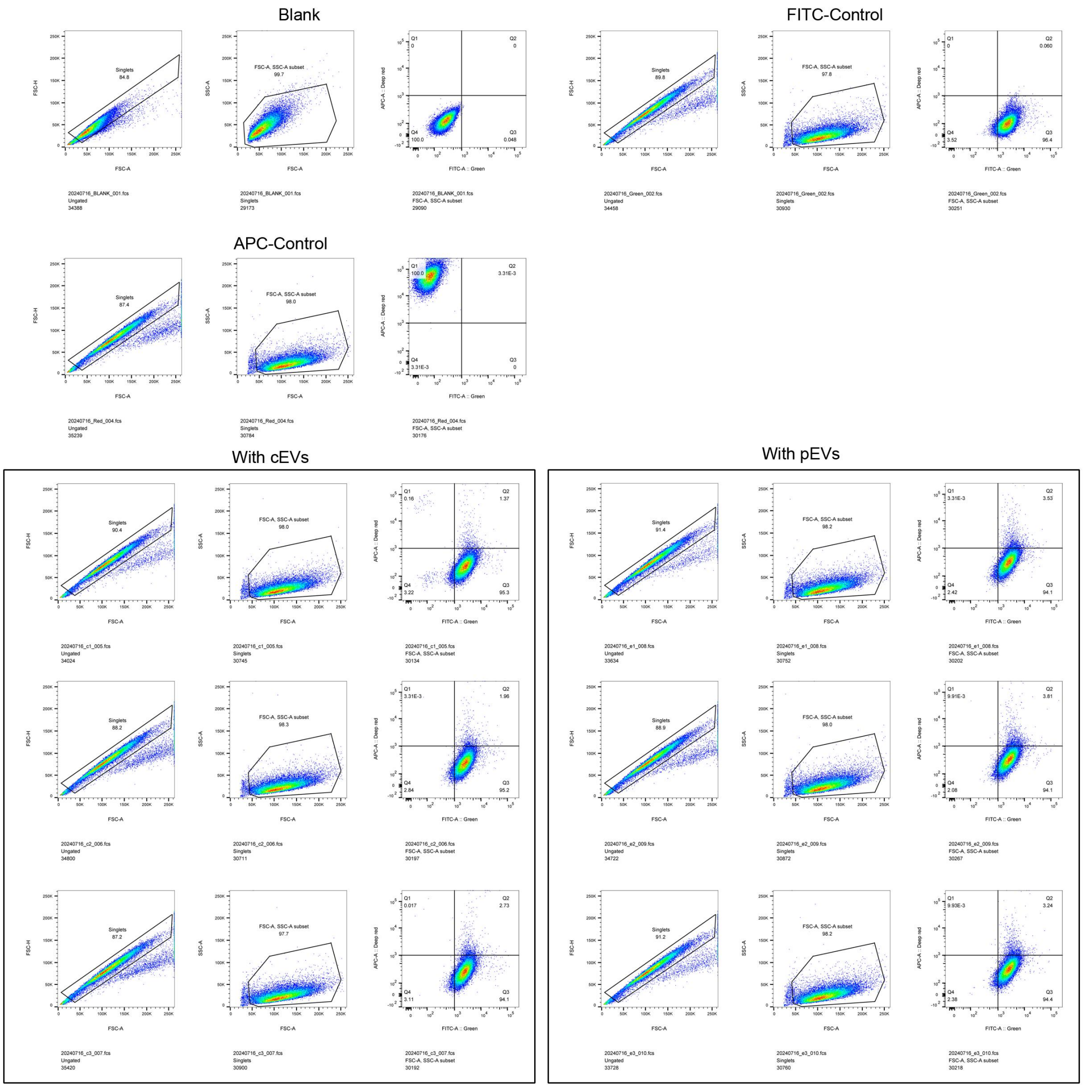

Figure S5. Gating strategies for analysis and percentage data of mitochondrial components transfer to HK-2 cells via cEVs or pEVs.

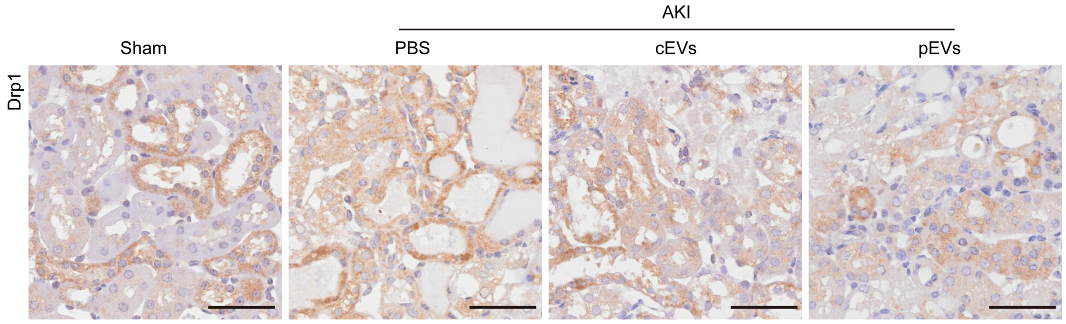

Figure S6. Expression of Drp1 in the AKI mice after cEVs and pEVs treatments. Scale bar, 50 μm.

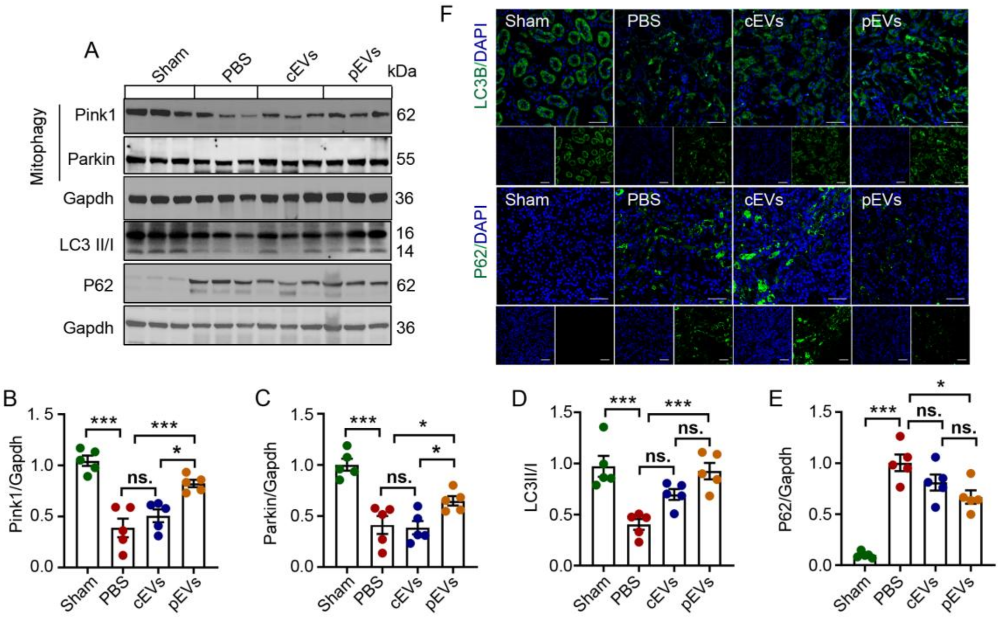

Figure S7. The pEV therapy demonstrated superior efficacy in restoring renal mitochondrial mitophagy. (A-F) Representative Western blot images (A) and quantitative analyses (B-E) of the mitochondrial mitophagy markers Pink1 (B), Parkin (C) and autophagy markers LC3 II/I (D), P62 (E). (F) Representative immunofluorescence images of LC3B and P62 in the kidney from AKI mice treated with cEVs or pEVs. Data are presented as means ± SEM, n = 5; $*p < 0.05$, $**p < 0.01$, $***p < 0.001$. ns, not significant. Scale bar, 50 μm.

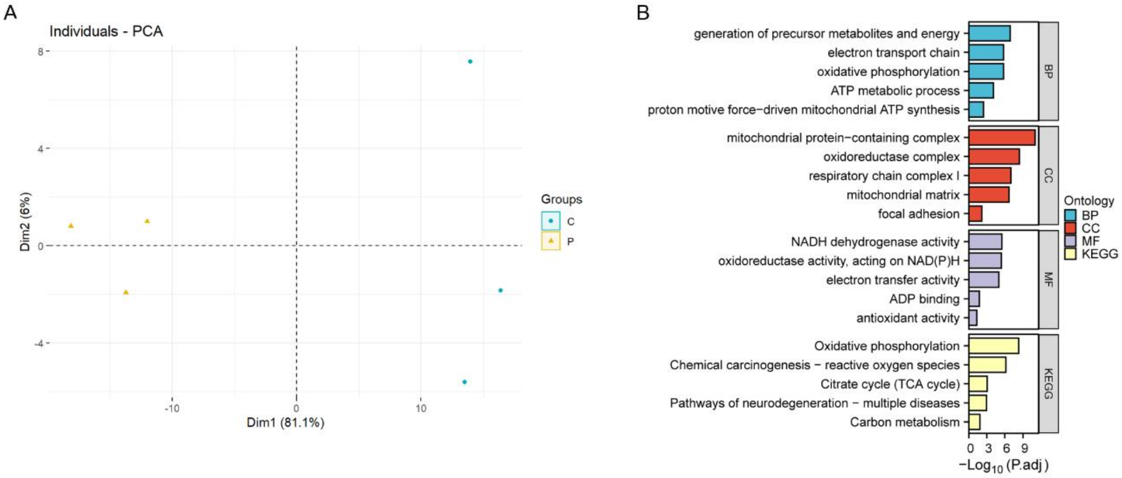

Figure S8. Identification and function enrichment of differentially expressed proteins (DEPs) between cEVs and pEVs. (A) PCA analysis for cEVs and pEVs samples. (B) The GO terms and KEGG pathway enrichment analysis of the DEPs.

Table S1. Primer sequences for qPCR analysis.

Gene	Accession number	Nucleotide sequence (5' to 3')	Species
Ngal	NM_008491.1	F-atggccctgagtgtcatgtg<br>R-aactgatcgctccggaagtc	Mus musculus
γ-H2ax	NM_010436.2	F-tacctcactgccgagatcct<br>R-cttgttgagctcctcgtcgt	Mus musculus
Gsdmd	NM_026960.4	F-tgcgtgtgactcagaagacc<br>R-caaacaggtcatccccacga	Mus musculus
Il1b	NM_008361.4	F-gggctgcttccaaacctttg<br>R-aagacacaggtagctgccac	Mus musculus
